# Risk prediction of osteoporotic vertebral compression fractures in postmenopausal osteoporotic women by machine learning modelling

**DOI:** 10.3389/fmed.2025.1664219

**Published:** 2025-09-12

**Authors:** Xiao Sun, Pengrui Jing, Yuqing Yang, Haifu Sun, Wenxiang Tang, Jian Mi, Pengju Zong, Qi Yan, Huilin Yang, Yusen Qiao

**Affiliations:** ^1^Department of Orthopaedics, The First Affiliated Hospital of Soochow University, Suzhou, China; ^2^Department of Otorhinolaryngology, The First Affiliated Hospital of Soochow University, Suzhou, China; ^3^Department of Orthopaedics, The Second Affiliated Hospital of Soochow University, Suzhou, China

**Keywords:** osteoporotic vertebral compression fracture, percutaneous kyphoplasty, postoperative refracture, osteoporosis, postmenopausal women, machine learning

## Abstract

**Background:**

Osteoporosis in postmenopausal women is characterized by significant bone mass loss due to reduced oestrogen, leading to an increased risk of osteoporotic vertebral compression fractures (OVCF). Comprehensive risk prediction models for diagnosing and predicting fracture risk in this population are still lacking.

**Objective:**

This study aims to identify key risk factors for OVCF in postmenopausal osteoporotic women and develop a machine learning model to predict OVCF risk by integrating clinical, biological, and musculoskeletal data.

**Methods:**

This retrospective case-control study included 486 postmenopausal women diagnosed with osteoporosis between 2015 and 2018. The patients were divided into a non-fracture group (Group A) and a vertebral fracture group (Group B) based on whether they developed OVCF during the subsequent 5 years of treatment and follow-up. Univariate and multivariate logistic regression analyses were performed to identify independent risk factors for OVCF. Furthermore, a comprehensive risk prediction model was constructed using multiple machine learning algorithms.

**Results:**

Among the 486 postmenopausal women, 269 (55.35%) experienced OVCF. Low bone mineral density (BMD), chronic inflammation, and sarcopenia were identified as independent risk factors, while regular anti-osteoporotic treatment was associated with a reduced fracture incidence. The Balanced Bagging machine learning model demonstrated an accuracy of 98.98%, a sensitivity of 98.24%, a specificity of 100.00%, and the model’s *F*_1_-score was 0.99. The deployed model outputs calibrated, patient-specific probabilities with case-level explanations and supports dynamic re-scoring as new BMD/CTx/NLR results become available, enabling personalized risk management in routine care.

**Conclusion:**

The development of OVCF in postmenopausal osteoporotic women is influenced by a combination of bone metabolism, inflammatory processes, and muscle health. The machine learning model developed in this study provides a reliable and accurate tool for personalized OVCF risk prediction, allowing clinicians to optimize prevention and treatment strategies. Future large-scale prospective studies are required to validate these findings and enhance the model’s predictive capabilities.

## Highlights

- Low bone mineral density (BMD), chronic inflammation (elevated NLR), and sarcopenia are identified as key independent risk factors for OVCF, while regular anti-osteoporotic treatment was associated with a reduced fracture incidence.
- The Balanced Bagging machine learning model integrates clinical, biological, and musculoskeletal data to predict osteoporotic vertebral compression fracture (OVCF) risk in postmenopausal women with 98.98% accuracy, 98.24% sensitivity, 100.00% specificity and the model’s *F*_1_-score was 0.99.
- Comprehensive multidimensional anti-osteoporosis therapy is expected to significantly reduce the incidence of OVCF, emphasizing the importance of comprehensive and multidimensional treatment of high-risk populations.

## 1 Introduction

Osteoporosis is characterized by reduced bone mass and disruption of bone microarchitecture. In postmenopausal women, oestrogen decline accelerates bone resorption, lowering bone mineral density (BMD) and increasing fragility fracture risk (1, 2). Among them, OVCF is one of the most frequent osteoporotic fractures (3). These fractures often occur in the thoracolumbar spine, leading to significant somatic pain and activity limitation, increasing the risk of disability and affecting patients’ quality of life (4, 5). Therefore, identifying the risk causes for OVCF in postmenopausal osteoporotic women and reducing the probability of fracture through effective preventive and therapeutic means are of great humanistic and socioeconomic importance. Despite numerous studies examining the relationship between osteoporosis and fragility fractures (4–8), the factors are not comprehensive enough to accurately assess the way they interact. Although BMD is widely used to diagnose osteoporosis and estimate fracture risk, reliance on BMD alone is insufficient because risk is also influenced by bone quality, systemic inflammation and bone turnover (9, 10). Moreover, the specific determinants of OVCF in postmenopausal women remain incompletely defined, and few studies have integrated diverse dimensions of risk in this population (11–14).

Bone turnover markers, including type I procollagen N-terminal propeptide (P1NP; formation) and carboxy-terminal cross-linked telopeptide of type I collagen (CTx; resorption), reflect skeletal metabolism, while osteocalcin (BGP) indicates osteoblastic activity (14–17). Calcium (Ca), phosphorus (P) and vitamin D3 (Vit D3) contribute to bone health maintenance, and clinical factors such as nutrition, hypertension and diabetes mellitus may modify OVCF risk (18, 19). Low-grade inflammation, captured by the neutrophil-to-lymphocyte ratio (NLR) and the systemic immune-inflammation index (SII), may potentiate bone resorption and fracture susceptibility (20). Emerging evidence links sarcopenia to higher vertebral fracture risk and poorer recovery. Postmenopausal women show greater age- and sex-related losses in muscle mass and strength, with more fatty infiltration of paraspinal muscles, supporting a role for muscle status in OVCF susceptibility. We therefore assessed the psoas major and multifidus muscle indices (PMI/MMI) as imaging surrogates (21, 22). Future work incorporating muscle quality and functional measures may clarify the incremental contribution of sarcopenia in postmenopausal osteoporosis. Against this background, we developed a machine-learning model that integrates clinical variables, densitometry, bone-turnover markers, inflammatory indices, and muscle metrics to identify the risk factors for OVCF and explore the correlations among these factors, thereby enabling personalized risk prediction and informing targeted prevention and treatment strategies (23, 24).

## 2 Methods

### 2.1 Management of the sample

The study protocol was approved by the Ethics Committee of the First Affiliated Hospital of Soochow University (2024 Lun Research Grant No. 728). This study investigated factors associated with osteoporotic vertebral compression fractures (OVCF) in postmenopausal women with osteoporosis. We analyzed 486 postmenopausal women diagnosed with primary osteoporosis between January 2015 and December 2018, all of whom received up to 5 years of outpatient follow-up. Patients were categorized into two groups based on the occurrence of OVCF. Inclusion criteria were: postmenopausal women with a menopause duration of 1–20 years, a diagnosis of primary osteoporosis, and regular follow-up for at least 5 years. Exclusion criteria included severe congenital or acquired spinal deformities, conditions significantly affecting bone metabolism, and incomplete medical or imaging records. Among the diseases that significantly affect bone metabolism are (1) chronic kidney disease (eGFR <60 mL^−1^ min^−1^). (2) Hyper- or hypoparathyroidism and thyroid dysfunction. (3) Chronic liver disease (Child–Pugh B/C). (4) Malabsorption syndrome. (5) Active malignancy or recent chemotherapy/radiotherapy. (6) Long-term systemic glucocorticoid therapy (≥5 mg prednisolone >3 months), aromatase inhibitors, antiepileptic drugs, heparin/warfarin. (7) Severe rheumatoid arthritis or other inflammatory arthritis. (8) Chronic alcohol abuse.

### 2.2 Observation indicators

We retrospectively collected demographic data, laboratory results, and imaging information, including age, BMI, fracture history, histories of hypertension and diabetes, serum calcium, vitamin D3, osteocalcin (BGP), type I collagen amino-terminal propeptide (P1NP), carboxy-terminal cross-linked telopeptide (CTx), bone mineral density (BMD), neutrophil-to-lymphocyte ratio (NLR), psoas major index (PMI), and multifidus muscle index (MMI). All patients underwent serological tests and dual-energy X-ray absorptiometry (DEXA) at diagnosis and during follow-up to assess BMD and bone metabolism markers. Time 0 was defined as the date of the index DXA. A standardised baseline assessment pathway was implemented, whereby bone turnover markers and lumbar MRI (for PMI/MMI) were obtained routinely on the same day as DXA whenever feasible, and otherwise within four weeks. Osteoporosis was defined according to the World Health Organization (WHO) criteria using dual-energy X-ray absorptiometry (DXA) at the lumbar spine (L1–L4) or the total hip. A *T*-score of ≤−2.5 indicates osteoporosis. In addition, a *T*-score between −1.0 and −2.5, in conjunction with fragility fractures of the pelvis and lumbar spine, can also serve as diagnostic criteria for osteoporosis. Given that this study focuses on vertebral fragility fractures caused by decreased bone density, and based on prior research and clinical experience, we set a *T*-score of <−1.5 as the criterion for sample inclusion.

Anti-osteoporosis medications at baseline and during follow-up (bisphosphonates, denosumab, teriparatide, or SERMs) were extracted from the electronic record and summarised in Table 1. Regimens followed product labelling (denosumab 60 mg SC every six months; teriparatide 20 μg SC daily) with vitamin D and calcium supplementation. BMD was reassessed at least annually (six-monthly in selected cases). Incident OVCF events were identified from the electronic record and confirmed radiographically using predefined criteria. The case records documented that participants underwent scheduled annual lateral thoracolumbar radiographs and symptom-triggered imaging to identify incident vertebral fractures. An incident vertebral fracture was defined according to Genant semi-quantitative criteria, or as a ≥20% and ≥4 mm reduction in vertebral body height (anterior, middle, or posterior) on follow-up compared with the most recent negative radiograph. Cases involving high-energy trauma were excluded, and each patient underwent lumbar magnetic resonance imaging (MRI) within 4 weeks of a radiograph showing a lumbar fracture to confirm the diagnosis.

**Table 1:** General data comparison between No vertebral fracture group (Group A) and Osteoporotic vertebral compression fracture group (Group B) patients.

	Group A (*n* = 217)	Group B (*n* = 269)	*p*
Age [mean (SD)]	62.68 (5.29)	63.58 (3.86)	0.036
Weight [mean (SD)]	49.32 (8.53)	50.69 (7.00)	0.057
BMI [mean (SD)]	24.66 (4.71)	25.63 (5.64)	0.045
History of fracture (%)			0.001
Yes	14 (6.45)	74 (27.51)	
No	203 (93.55)	195 (72.49)	
Hypertension (%)			0.153
Yes	20 (9.22)	36 (13.38)	
No	197 (90.78)	233 (86.62)	
Diabetes (%)			0.031
Yes	7 (3.23)	21 (7.81)	
No	210 (96.77)	248 (92.19)	
BMD [mean (SD)]	−2.56 (0.37)	−2.79 (0.31)	0.001
Osteoporosis treatment (%)			0.011
Yes	184 (84.79)	203 (75.46)	
No	33 (15.21)	66 (24.54)	
Albumin [mean (SD)]	38.32 (7.71)	37.29 (5.52)	0.990
Calcium [mean (SD)]	2.36 (0.23)	2.32 (0.17)	0.027
Phosphorus [mean (SD)]	1.16 (0.17)	1.13 (0.21)	0.190
VitD3 [mean (SD)]	24.61 (6.71)	17.86 (7.27)	0.001
BGP [mean (SD)]	26.28 (8.52)	19.95 (8.98)	0.001
CTx [mean (SD)]	215.71 (182.05)	713.15 (329.80)	0.001
P1NP [mean (SD)]	33.02 (20.51)	75.14 (87.01)	0.001
NLR [mean (SD)]	1.98 (0.58)	2.12 (0.92)	0.044
SII [mean (SD)]	385.37 (115.41)	417.59 (218.94)	0.038
PMI [mean (SD)]	4.07 (0.63)	3.91 (0.57)	0.003
MMI [mean (SD)]	19.38 (4.96)	18.78 (5.12)	0.197

SD, standard deviation; BMI: body mass index; BGP, osteocalcin; CTx, C-terminal telopeptide of type I collagen; P1NP, procollagen type I N-terminal propeptide; NLR, neutrophil-to-lymphocyte ratio; SII, systemic immune inflammation index; PMI, psoas muscle index; MMI, multifidus muscle index.

Additionally, cross-sectional areas of the psoas and multifidus muscles at the L3 level were measured on T2-weighted MRI, with PMI and MMI calculated by normalizing muscle area to height squared (cm^2^/m^2^). Sarcopenia was defined as a PMI below 3.9 cm^2^/m^2^. All images were independently reviewed by two fellowship-trained musculoskeletal radiologists who were blinded to the clinical predictors, with disagreements resolved by consensus.

### 2.3 Statistical analysis

Statistical analysis was performed using SPSS software (version 27.0, IBM, United States), presenting continuous data as mean ± standard deviation analyzed with independent *t*-tests. Categorical data were evaluated using chi-square tests or Fisher’s exact tests. A *p*-value <0.05 was considered significant. Variables that demonstrated significant differences in the univariate analysis were subsequently included in a multivariate logistic regression to identify independent risk factors for OVCF among postmenopausal women with osteoporosis. Prior to multivariable modelling, we assessed pairwise dependencies and applied a VIF threshold of <5.0 to evaluate collinearity. For clusters of biologically related predictors (e.g., SII with NLR; CTx with P1NP/BGP), we retained the clinically more parsimonious and statistically more robust variable (NLR and CTx, respectively). For transparency, BGP, P1NP, SII, and PMI were included in descriptive statistics and exploratory visualisations but were not carried forward into the comparative modelling process.

### 2.4 Model analysis

In this study, various machine learning models—including Logistic Regression, Naive Bayes, SVM, Decision Tree, AdaBoost, Gradient Boosting, and Balanced Bagging—were developed using Python 3.11.7 with the scikit-learn library. Since the retrospectively collected laboratory dataset contained only a small fraction of missing values, we employed the missForest algorithm, thereby minimizing information loss while preserving the underlying distribution structure. Model training employed 10-fold cross-validation and grid search for hyperparameter optimization: the former partitions the training data into 10 subsets, iteratively using nine for training and one for validation to reduce partition bias, while grid search systematically explores a predefined parameter space to identify the optimal parameter combination. Model performance was evaluated using metrics such as AUC, sensitivity, specificity, PPV, NPV, accuracy, *F*_1_-score, and Brier Score, ensuring a comprehensive assessment of classification ability, stability, and calibration. To control overfitting in high-variance learners, we imposed *a priori* constraints (for tree/boosting models: max_depth ≤3, min_samples_leaf ≥20, learning_rate ≤0.05) and used class-balanced resampling within a Balanced Bagging framework. Learning curve analyses showed that performance plateaued once the training sample exceeded approximately 200 cases; permutation tests (1,000 shuffles) yielded an AUC of approximately 0.50, indicating that the observed discrimination was not due to chance. Additionally, SHAP (SHapley Additive exPlanations) was applied to quantify each feature’s contribution to the model’s predictions, enhancing interpretability and providing insights into the decision-making process. The same individual can be re-scored at follow-up when new densitometry or laboratory results are available, allowing trajectory monitoring after treatment initiation or adjustment. A five-feature configuration (BMD T-score, CTx, NLR, age, BMI) preserved >90% of full-model discrimination, supporting deployment where selected assays are unavailable.

## 3 Result

### 3.1 Patient characteristics

A total of 486 postmenopausal osteoporotic women (mean age 63.18 ± 4.57 years) were included, with 217 in the non-fracture group (Group A) and 269 in the OVCF group (Group B). Significant differences between groups were observed in BMI (*p* = 0.045), fracture history (*p* = 0.001), BMD (*p* = 0.001), calcium (*p* = 0.027), Vit D3 (*p* = 0.001), and bone turnover markers (CTx and P1NP, *p* = 0.001) (Table 1).

### 3.2 Visualization analysis

**Figure 1:**
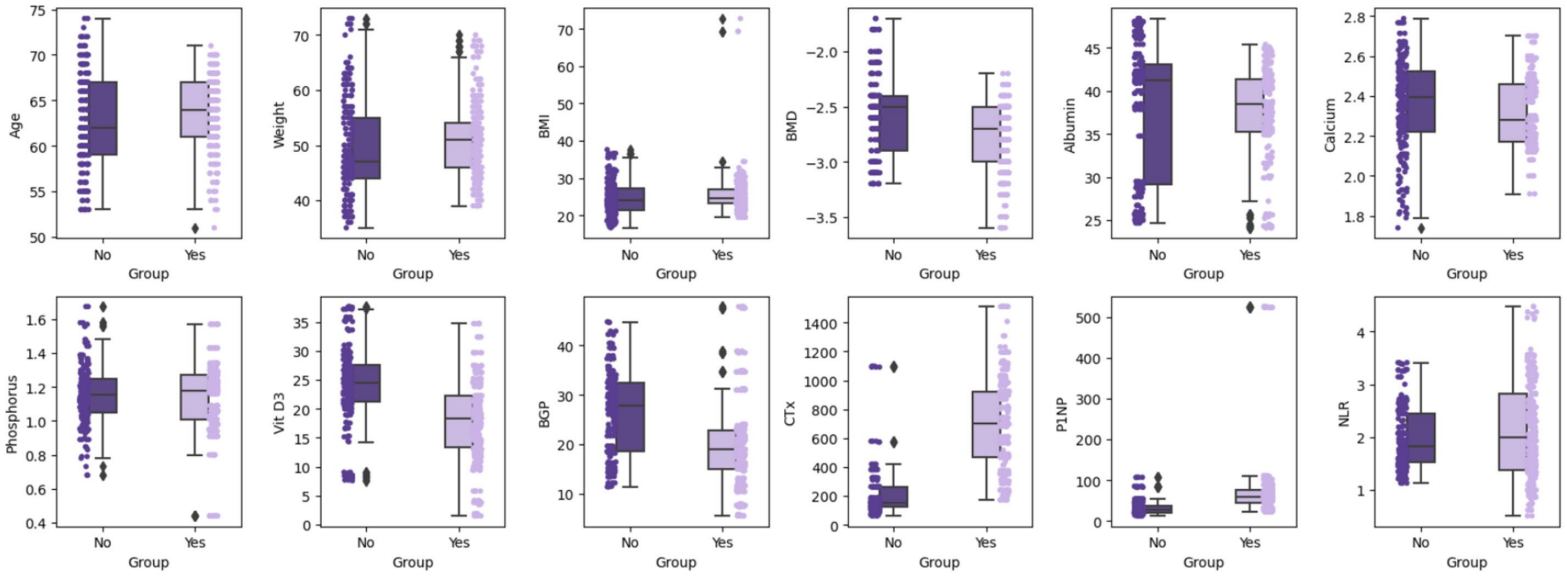
Box plot comparison of continuous variables by groups. Using box plots and scatter points, it displays the median, quartiles, and outliers of each variable in different groups, providing a visual comparison of the differences between these variables.

**Figure 2:**
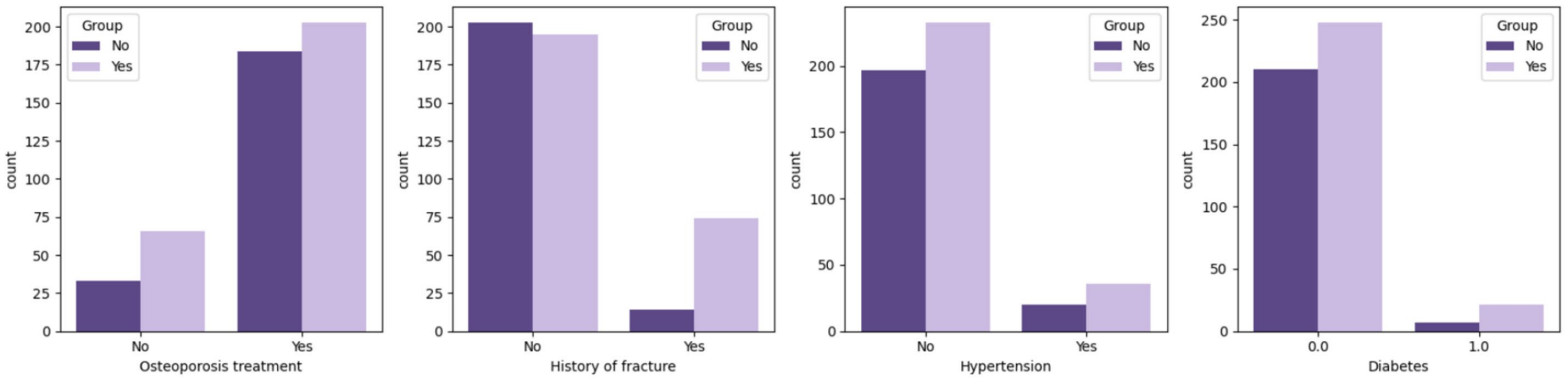
Categorical variable distribution between Group A and Group B. The graph shows the distribution of several categorical variables: treatment for osteoporosis, history of fracture, hypertension, and diabetes. The bar graph compares the counts for each category in these variables, providing a clear visual comparison of the frequency distribution between the two groups.

Box plots showed that Group B had significantly higher age and CTx levels, and lower BMD and Vit D3 levels (Figure 1). Bar charts revealed that Group B had a higher fracture history and lower osteoporosis treatment rates compared to Group A (Figure 2), suggesting that age, BMD, fracture history, and treatment status are key predictors of OVCF.

### 3.3 Univariate and multivariate analysis

Univariate analysis demonstrated significant associations (*p* < 0.05) between OVCF and variables such as age, BMI, fracture history, diabetes, BMD, osteoporosis treatment, calcium, Vit D3, CTx, P1NP, NLR, SII, and PMI. Multivariate logistic regression (Hosmer–Lemeshow *p* = 0.565) identified age, BMI, fracture history, BMD, osteoporosis treatment, calcium, Vit D3, CTx, and NLR as independent risk factors for OVCF (Table 2).

**Table 2:** Results of multi-factor logistic regression analysis of risk factors associated with osteoporotic vertebral compression fractures (OVCF) in postmenopausal women.

Influencing factor	*B*	OR	Tolerance	VIF	95% CI	*p*
Age	0.249	1.283	0.968	1.033	1.132–1.453	0.001
BMI	0.267	1.306	0.877	1.141	1.149–1.486	0.001
History of fracture			0.805	1.242		
No		Reference				
Yes	2.714	15.082			4.577–49.695	0.001
Diabetes			0.954	1.048		
No		Reference				
Yes	1.444	4.236			0.844–21.274	0.080
BMD	−5.515	0.004	0.842	1.187	0.001–0.026	0.001
Osteoporosis treatment			0.964	1.038		
No		Reference				
Yes	−1.507	0.221			0.072–0.678	0.008
Calcium	−3.297	0.037	0.904	1.106	0.005–0.281	0.001
VitD3	−0.237	0.789	0.688	1.453	0.722–0.862	0.001
BGP	−0.038	0.962	0.825	1.212	0.913–1.014	0.151
CTx	0.011	0.957	0.542	1.844	0.926–0.990	0.001
P1NP	0.017	0.521	0.544	1.838	0.319–0.851	0.225
NLR	0.780	2.182	0.521	1.920	1.046–4.552	0.037
SII	−0.003	0.997	0.539	1.856	0.993–1.001	0.101
PMI	−0.282	0.754	0.939	1.064	0.384–1.484	0.414

### 3.4 Multi-model predictive analysis

**Figure 3:**
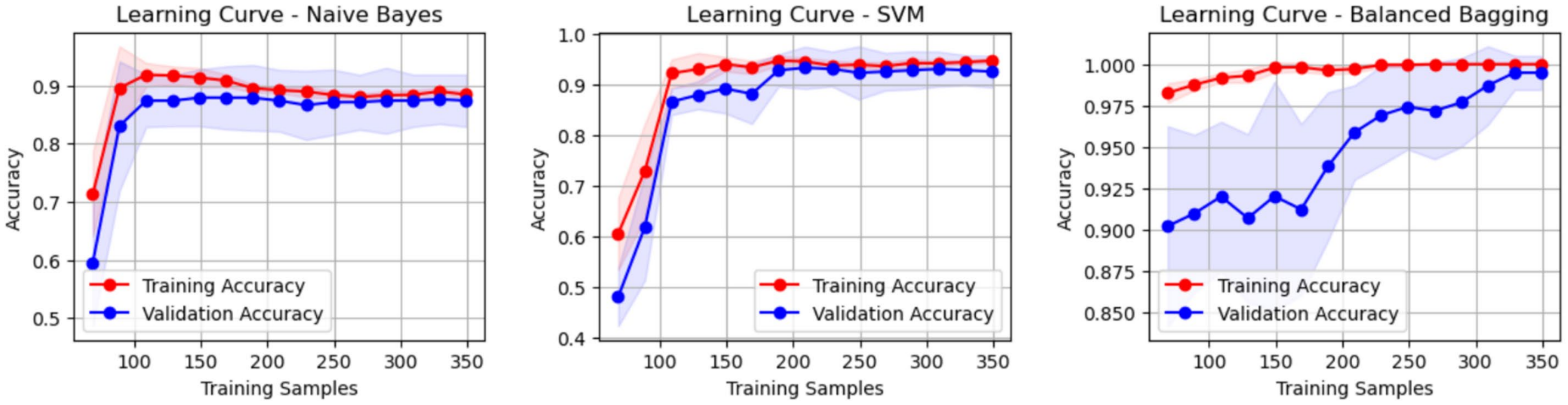
Internal validation results—cross-validation performance evaluation.

**Figure 4:**
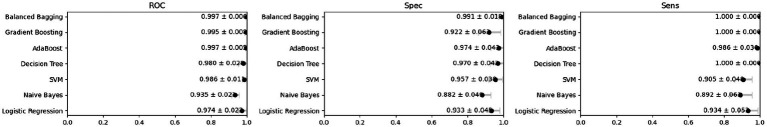
Learning curve analysis—training and validation accuracy trends.

**Figure 5:**
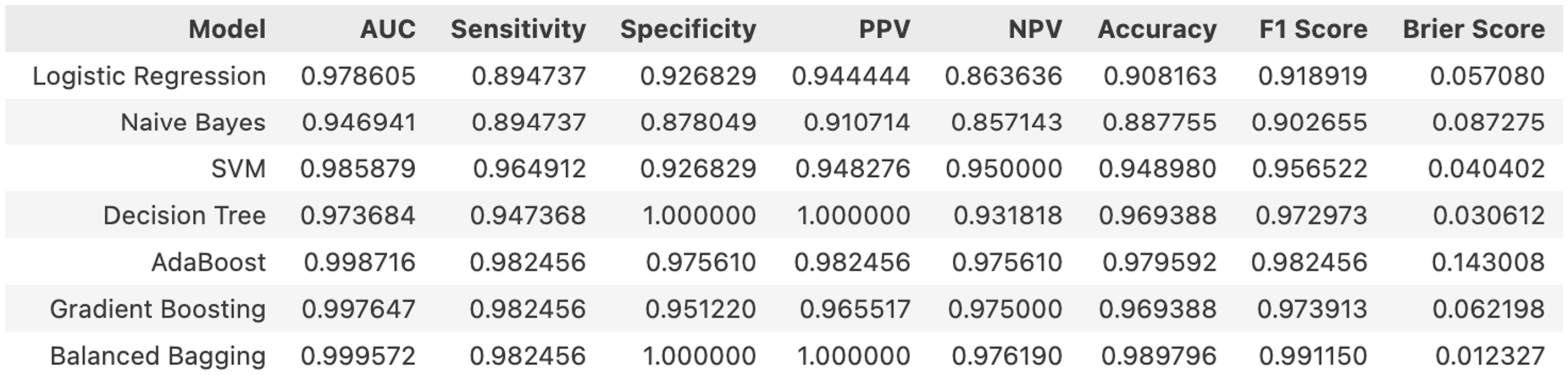
External validation results—generalization performance evaluation.

**Figure 6:**
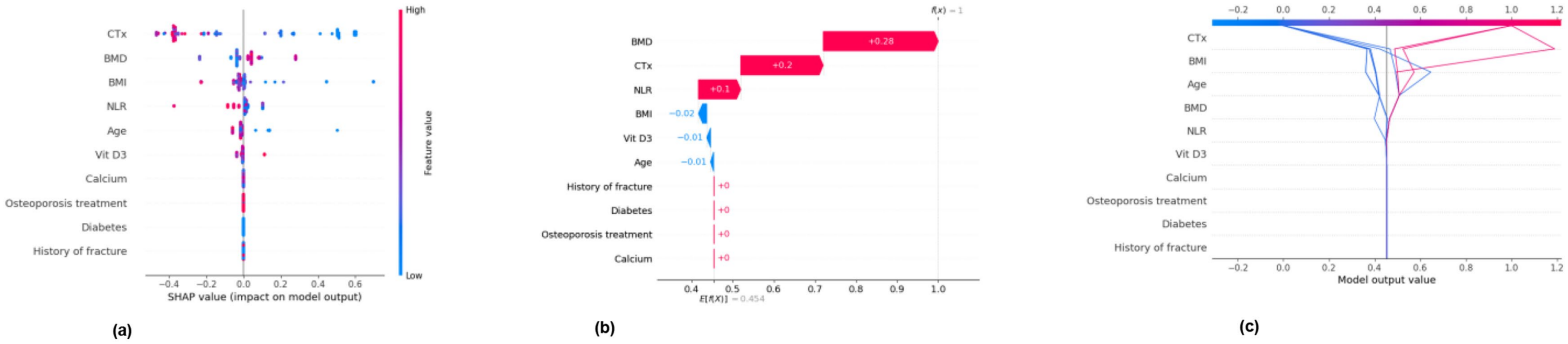
SHAP analysis (negative)—feature importance for negative class. **(a)** SHAP summary dot plot. **(b)** SHAP waterfall plot for the 11th sample. **(c)** SHAP decision plot.

**Figure 7:**
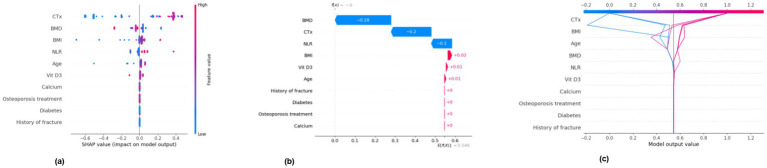
SHAP analysis (positive)—feature importance for positive class. **(a)** SHAP summary dot plot. **(b)** SHAP waterfall plot for the 11th sample. **(c)** SHAP decision plot.

Internal cross-validation (Figures 3, 4) showed that model performance stabilized with over 200 training samples. On the external dataset (Figure 5), the Balanced Bagging model outperformed others, achieving an AUC of 0.9956 and an accuracy of 98.98%, with high sensitivity and specificity. SHAP analysis (Figures 6, 7) indicated that CTx, BMD, BMI, NLR, age, and Vit D3 were the most influential features—particularly CTx, BMD, and NLR—in discriminating between classes. Overall, the model effectively captured multidimensional patterns, supporting robust OVCF risk prediction.

## 4 Discussion

In this single-centre cohort of 486 postmenopausal women, we identified age, BMI, prior fracture, lower BMD, absence of anti-osteoporotic treatment, lower calcium, lower Vit D3, higher CTx and higher NLR as independent correlates of OVCF, reflecting the interplay of skeletal fragility, bone turnover and systemic inflammation (25). Our machine-learning analysis, using these nine features, achieved excellent discrimination and calibration; Balanced Bagging performed best (temporal hold-out AUC 0.9956; accuracy 98.98%), and learning curves plateaued beyond approximately 200 cases, supporting model stability. Brier score and calibration plots indicated well-calibrated probabilities. SHAP analyses consistently ranked CTx, BMD and NLR as the most influential contributors, with age, BMI and Vit D3 providing additional signal.

OVCF is the most prevalent complication of osteoporosis in postmenopausal women, typically affecting the thoracolumbar spine and causing pain, functional limitation and healthcare burden (26–29). Lower BMD remained a dominant risk factor in our data, consistent with its status as the diagnostic standard and a core determinant of vertebral fragility. Reduced mineral content is accompanied by deterioration of microarchitecture (trabecular thinning, loss of connectivity and endplate microcracks), decreasing load-sharing capacity and raising the likelihood of wedge or biconcave deformities under routine activities (30–33). CTx, a marker of type I collagen degradation, reflects systemic osteoclastic activity. Elevated CTx signifies uncoupled remodelling with increased resorption cavities, transient porosity and weaker bone packets before secondary mineralisation is complete (34, 35). In the vertebral body—rich in trabecular bone—this manifests as reduced stiffness and earlier yield under axial load. CTx outperformed formation markers (P1NP, BGP) for discrimination, implying that resorption acceleration, rather than formation dynamics, is the proximate driver of vertebral failure in this setting. SHAP showed monotonic risk escalation with rising CTx, supporting a dose-response relation. Chronic low-grade inflammation likely amplifies this risk: oestrogen decline is associated with increased pro-inflammatory cytokines (e.g., TNF-α, IL-6), which elevate NLR, suppress osteoblastic activity and enhance osteoclastic activity, thereby weakening bone structural strength (36–40). BMI showed a positive association with OVCF, a finding compatible with sarcopenic obesity and visceral adiposity, which promote systemic inflammation despite higher absolute mass; this may offset any mechanical protection conferred by weight alone (41). Although PMI was lower in the OVCF group at the univariate level, it did not remain independent after adjustment, suggesting that global anthropometry and biochemical drivers captured the incremental risk more parsimoniously in this dataset.

Among tested learners, the Balanced Bagging model yielded the lowest cross-validated deviance with favorable calibration, likely because bagging reduces variance under class imbalance and captures non-linear interactions without overfitting when depth and leaf size are constrained (30). The Balanced Bagging framework can be retrained with reduced feature sets, allowing deployment when certain biomarkers (e.g., CTx) are temporarily unavailable. SHAP analyses show that a core subset—BMD, CTx, NLR, age, BMI—retains >90% of the model’s discriminative capacity, supporting a “tiered” prediction approach adaptable to real-world data completeness. These findings support a multidomain approach to risk stratification that integrates densitometry, resorption activity and inflammatory tone. Embedded within electronic health records, the model can deliver patient-specific risk with SHAP-based explanations, guiding optimisation of anti-resorptive therapy, calcium-vitamin D repletion and resistance exercise, and prioritising high-risk patients for early intervention (36, 42). Our findings suggest that the combination of bone-specific translational markers (especially CTx), systemic inflammation index (NLR), and lumbar paravertebral muscle health index (PMI) can lead to a multifactorial and comprehensive assessment model of skeletal vulnerability.

In clinical applications, machine learning-based risk prediction tools can help physicians identify at-risk individuals at an early stage and implement personalized interventions for treatment. By comprehensively evaluating individual patients, the model can be embedded into electronic health record systems, providing clinicians with a more intuitive risk assessment to identify women with osteoporosis whose biochemical or imaging parameters indicate fracture vulnerability. Helps develop more effective prevention and treatment strategies. Comprehensive factor analysis helps identify patient deficiencies and provide multidimensional interventions. Early intensive anti-osteoporosis treatment, optimization of calcium-vitamin D status, and targeted resistance training can effectively stop the transition from osteoporosis to bone loss, thereby preventing the first OVCF. For future clinical deployments, our strategy is to reduce the need for bespoke data collection by leveraging data generated from routine care and providing a calibrated, patient-specific probability at the point of care. The production model uses nine routine variables, and a five-feature configuration (BMD T-score, CTx, NLR, age, BMI) retains >90% of the full model’s discriminative power. The tool remains deployable in resource-limited settings, while federated updating and periodic recalibration support sustainable, privacy-preserving model improvement across hospitals. Unlike single-modality calculators (e.g., DEXA thresholds or turnover markers alone) or physics-only morphometric models, our framework integrates densitometry, biochemical resorption activity, inflammatory tone, and clinical context to produce calibrated, patient-specific probabilities with case-level explanations. This multimodal, EHR-embeddable approach supports targeted, modifiable interventions and remains deployable in resource-limited settings via a five-feature configuration that preserves >90% of full-model discrimination.

Although this study explored the risk factors for OVCF in postmenopausal women with osteoporosis through multifactorial analysis and machine learning modelling, there are still some limitations. First, this study was a retrospective study with a single sample source. Future large-scale prospective studies are needed to validate the broad applicability of these risk factors and predictive models. All participants in the study were recruited from China; therefore, genetic backgrounds, lifestyle factors, and medical care may differ from other ethnic groups, which may not make the model generalisable worldwide. Future prospective multicentre validation in European and North American populations will be sought, as well as recalibration of model thresholds for different ethnic backgrounds. In addition, given the low prevalence and incomplete ascertainment of some secondary causes of altered bone metabolism, we treated these factors as exclusion criteria rather than including them as covariates in the model. As a result, our model may not be generalisable to these subgroups and could potentially underestimate the risk in such contexts. Thirdly, although the baseline calibration was favorable, the time-varying treatment exposure (osteoporosis treatment regimen) and adherence were not explicitly modeled. Future studies should explore the influence of more potential factors on secondary OVCF in postmenopausal osteoporotic women to further improve the prediction model.

## 5 Conclusion

This study identifies low BMD, chronic inflammation, and bone breaking indicator as key risk causes for osteoporotic vertebral compression fractures (OVCF) in postmenopausal women, while regular anti-osteoporotic treatment reduces fracture risk. Multiple machine learning developed provides a reliable tool for personalized OVCF risk prediction, integrating clinical, biological, and musculoskeletal data to enhance prevention and treatment strategies. Future studies are needed to validate the model and explore additional factors, improving its accuracy and clinical application.

## Data Availability

The original contributions presented in the study are included in the article/supplementary material, further inquiries can be directed to the corresponding authors.
